# Health economic evaluation in orthotics and prosthetics: a systematic review protocol

**DOI:** 10.1186/s13643-019-1066-9

**Published:** 2019-06-27

**Authors:** Leigh Clarke, Michael Dillon, Alan Shiell

**Affiliations:** 1 Discipline of Prosthetics and Orthotics, Department of Physiotherapy, Podiatry, Prosthetics and Orthotics, School of Allied Health, Human Services and Sport, La Trobe University, Melbourne, Victoria 3083 Australia; 2 The Australian Orthotic Prosthetic Association Ltd, Melbourne, Victoria 3124 Australia; 3 Department of Public Health, School of Psychology and Public Health, La Trobe University, Melbourne, Victoria 3083 Australia

**Keywords:** Systematic review, Economic evaluation, Health economics, Cost-effectiveness, Cost-benefit, Orthotics, Prosthetics

## Abstract

**Background:**

Health economic evaluations are essential to support health care policy and investment decisions. To date, health economic evaluations in orthotics and prosthetics have focused on discrete components of an orthosis/prosthesis (e.g. a microprocessor controlled prosthetic knee joint) rather than the broader service provided by orthotist/prosthetists. As such, the contribution to orthotic/prosthetic policy and investment decisions is unclear. Whilst there are opportunities to conduct more informative health economic evaluations that describe the costs and benefits of the orthotic/prosthetic service, it is important that prospective research is informed by a critical review of the method design challenges and an understanding of how this research can be improved. The aim of this systematic review is to critically appraise the existing orthotic/prosthetic health economic evaluation literature and therefore determine evidence gaps, critical method design issues and the extent to which the literature informs orthotic/prosthetic policy and investment decisions.

**Methods:**

A comprehensive range of databases—AMED, EMBASE, MEDLINE and PsychINFO, Cumulative Index of Nursing and Allied Health Literature (CINAHL), ProQuest Nursing and Allied Health, Web of Science, Cochrane Database of Systematic Reviews (CDSR) and specialty health economic databases—will be searched using National Library of Medicine Medical Subject Headings (MeSH) terms as well as the title, abstract, and keyword terms. Search terms related to the intervention (e.g. orthosis), including variants used by varying professional disciplines (e.g. brace), will be used in preference to defining the populations that use orthotic and prosthetic services (e.g. people living with rheumatoid arthritis). Search terms related to health economic evaluations will be guided by previously developed and tested search strings and align with recommendations by the Canadian Agency for Drugs and Technologies in Health. Articles meeting the inclusion criteria will be hand-searched for relevant citations, and a forward citation search using Google Scholar will also be conducted to identify early online articles not yet indexed in traditional databases. Original research published in the English language and after 1 January 2000 will be included. The Checklist for Health Economic Evaluation Reporting Standards (CHEERS) and the Consensus on Health Economic Criteria (CHEC)-Extended list will be used to appraise the methodological quality and identify sources of bias. Data extraction and appraisal will be conducted by one reviewer independently using appraisal instrument guidelines and a content specific decision aid with exemplars. A subsequent review by a second researcher will be undertaken to confirm the accuracy of the extraction and appraisal, and a final review by a third where consensus cannot be reached. The data will be extracted to a purpose-built data extraction template with decision-making guidelines to support consistency. Where possible, the findings of the review will be reported as a meta-analysis, although the heterogeneity of the literature will likely mean a narrative review that illuminates method design issues that contribute to imprecision and variation will be more appropriate.

**Discussion:**

This protocol has been purposefully designed to summarise the existing evidence and appraise the methodological approaches used and the quality of the health economic evaluations in orthotics and prosthetics. What we learn from this review will be used to guide further work in this area and design more rigorous health economic evaluations into the future.

**Systematic review registration:**

PROSPERO CRD42018116910.

**Electronic supplementary material:**

The online version of this article (10.1186/s13643-019-1066-9) contains supplementary material, which is available to authorized users.

## Background

People with mobility-related impairments, such as stroke, cerebral palsy or amputation, use orthoses/prostheses to assist with function, activity and participation [1].

Historically, orthoses/prostheses were provided by highly skilled tradespeople with little responsibility beyond the manufacture and supply of the orthosis/prosthesis [2–4], and as such, there was significant medical oversight for the associated clinical role which included prescription and reviews [2].

Over time, the role of the orthotist/prosthetist has become increasingly clinical [2, 5] as education and training have shifted from an apprenticeship model to tertiary education that emphasised the clinical role necessary for autonomous professional practice [3]. As such, many aspects of the clinical role that were once provided by medical specialists are now the responsibility of the orthotist/prosthetist including prescription, reviews and education, as illustrative examples [3, 5].

Into the future, the clinical role of the orthotist/prosthetist will continue to evolve with the introduction of new, contemporary models of care that focus on supporting clients to identify the goals of their treatment [6] and measure how successfully these goals have been achieved. By way of example, the Australian National Disability Insurance Scheme, a national funding model for disability services and interventions, has been designed to redress the historical, paternalistic prescribing role of practitioners [7]. Instead, practitioners are expected to use high-level communication skills to facilitate discussions, thereby supporting clients to identify their own treatment goals [6, 8] and inform their decision about orthotic/prosthetic interventions given an understanding of the likely outcomes [7, 9]. Practitioners are also expected to evaluate the effectiveness of an intervention using valid and reliable outcome measures [10, 11] and, in this way, demonstrate the contribution that the intervention makes towards the attainment of a client’s goals.

The enhanced clinical role of the orthotist/prosthetist and the introduction of more contemporary models of care have fundamentally changed the way orthotic/prosthetic clinical services are provided. However, the small pool of orthotic/prosthetic health economic evaluation literature has remained focussed on evaluating the cost-effectiveness of newer, high-cost technologies (e.g. microprocessor prosthetic knees or bone-anchored prostheses) compared to the current standard technologies [12–17]. Whilst these studies make a meaningful contribution to technology-related policy decisions, they do not inform decisions regarding the funding of comprehensive clinical services provided by orthotist/prosthetists that focus on supporting clients to achieve their goals of which the provision of technology is a component.

A prudent first step to developing a contemporary approach to orthotic and prosthetic health economic evaluations would be to conduct a systematic review of the current orthotic/prosthetic literature. Critical appraisal of the available health economic evaluation research would identify evidence gaps and critical method design issues that could inform an assessment of the extent to which current literature can meaningfully contribute to decisions about funding of contemporary clinical services of which provision of an orthosis/prosthesis is part. The outcomes of the systematic review could also inform the design of more rigorous health economic evaluations into the future.

In summary, health economic evaluations are essential to support health care policy and investment decisions. To date, health economic evaluations in orthotics and prosthetics have focused on discrete components of an orthosis/prosthesis (e.g. a microprocessor-controlled prosthetic knee joint), and whilst these studies can help inform technology-related policy decisions, they are unlikely to be well-designed to inform decisions about contemporary clinical services in which technologies, such as prostheses and orthoses, are provided. Therefore, it is unclear whether current evaluations adequately contribute to cost-effectiveness knowledge, policy and investment decisions for orthotic/prosthetic services. Into the future, there are opportunities to conduct health economic evaluations where the costs and benefits of the orthotic/prosthetic service are measured in contemporary healthcare models, such as the National Disability Insurance Scheme, therefore informing health care policy and investment decisions for people requiring orthoses/prostheses within such schemes. A prudent first step would be to conduct a systematic review of the current orthotic/prosthetic health economic literature to identify evidence gaps, and method design issues that can inform the design of rigorous health economic evaluations focused on the cost-effectiveness of contemporary services of which the provision of a prosthesis/orthosis is part.

The aim of this systematic review is to critically appraise the existing orthotic/prosthetic health economic evaluation literature and therefore determine evidence gaps, critical method design issues and the extent to which the literature informs orthotic/prosthetic policy and investment decisions.

## Method

### Search strategy

The OVID platform will be used to search the following databases independently: AMED, EMBASE, MEDLINE and PsychINFO. Separate searches will also be conducted for Cumulative Index of Nursing and Allied Health Literature (CINAHL), ProQuest Nursing and Allied Health, Web of Science and the Cochrane Database of Systematic Reviews (CDSR). In line with recent search strategy recommendations, core database searches will be complemented with health economic specific database searches including Centre for Reviews and Dissemination (CRD) Health Technology Assessment and the National Health Service Economic Evaluation Database (NHS EED) [18–21].

National Library of Medicine Medical Subject Headings (MeSH) will be used for the relevant aforementioned databases, including the terms for *Artificial Limbs*, *Amputation*, *Amputees*, *Orthotic Device*, *Cost-benefit Analysis*, *Costs and Cost Analyses* and *Quality-adjusted Life Years*. These MeSH terms include all relevant root and hierarchical branches related to the intervention (i.e. *prostheses* and *orthoses*) and outcome (e.g*. health economic evaluation*, *cost-effectiveness*) and will also be exploded to reduce the likelihood that relevant publications will be overlooked due to variations in the way articles are indexed.

Search terms related to the intervention (i.e. *prostheses* and *orthoses*), as well as variants used by varying professional disciplines (e.g. *splint*, *brace*), will be used in preference to defining the populations that use orthotic and prosthetic services which are very broad (e.g. *cerebral palsy*, *stroke*, *scoliosis*, *rheumatoid arthritis*, *plagiocephaly*). Search terms related to health economic evaluations (e.g. *cost-effectiveness*, *cost-utility*) will be based on previously developed and tested search strings [22, 23] and align with recommendations by the Canadian Agency for Drugs and Technologies in Health [20].

Synonyms and acronyms for each of the aforementioned search terms will be used in combination with Boolean operators and wild cards to ensure variations in spelling and punctuation (e.g. *cost effectiveness* and *cost-effectiveness*) are captured. Search terms and larger search strings will be developed, tested and modified based on the search results from each database that highlight terms in the title (ti), abstract (ab) and keyword (kw) that were relevant. Where these field codes vary across databases, the appropriate alternative will be used.

Search terms designed to exclude irrelevant literature will also be used given a large body of research relating to internal or dental prostheses (e.g. hip or dental implants). In this way, studies pertaining to internal hip and knee replacements and other non-limb prosthetic results (e.g. aortic, valve, breast) will be excluded. It is known that dental literature uses numerous terms (e.g. *overdentures*, *implant-supported*, or *restoration*) that are also non-dental specific (e.g. *implant*) therefore preventing efficient exclusion using single search terms and will inadvertently exclude relevant prosthetic literature (e.g. osseointegration studies in which the term implant is used). Therefore, instead of using search terms to exclude irrelevant dental literature, a MeSH search strategy will be used (i.e. *Exp Dentistry/*), allowing exclusion of all studies indexed under this subject heading, including all root and hierarchical branches.

The aforementioned MeSH and search terms were developed through an analysis of the keywords, titles and abstracts of known relevant journal articles. As part of protocol development, and to ensure the rigour of this approach, testing was undertaken for one database. This included an assessment of the comprehensiveness and precision of the search yield against a pool of known orthotic and prosthetic health economic evaluation studies with wide-ranging publication years, keywords and journals. Where the search failed to identify known articles, the search terms and strings were refined and retested. The testing also allowed for confirmation of search methodology decisions, for example, the use of intervention only search term (i.e. exclusion of population terms) was found to be sufficient to identify a pool of known articles without defining the patient population and generic search terms related to quality of life (QoL) yielded substantial literature of a general nature, unrelated to health economic evaluation in testing, therefore confirming the reasonableness of its exclusion.

Given that the author’s native language is English and that restriction of non-English language articles does not alter the conclusions of systematic reviews and meta-analyses [24], all search results will be limited to studies published in the English language. The search results will also be limited by time (i.e. 1 January 2000 to end search date). Time limits were tested as part of search strategy development. We were unable to identify any orthotic and prosthetic literature that met the definition of a health economic evaluation published prior to the year 2000. This is due to the introduction of health technology assessment measures in the late 1990s (e.g. the National Institute for Health and Care Excellence (NICE) in the UK was established in 1999), which provided guidance for contemporary methodological approaches in the evaluation of new healthcare technologies, and therefore stimulated health economic evaluation research with a reported increase from 2007 to 2012 of 79% and 108% in the NHS Economic Evaluation Database (NHS HEED) and PubMed, respectively [21].

The search results will also be limited to original, peer-reviewed research and as such, commentaries and reviews will be excluded. As part of protocol development, the decision to exclude grey literature was tested. We identified grey literature that met the operational definition of a health economic evaluation; however, they were concurrently published in peer-review journals and, as such, the journal publication provided a stronger form of evidence (e.g. Dobson DaVanzo & Associates Report 2013 and the Queensland Artificial Limb Scheme Report 2018 are also peer-review published) [14, 25–27]. This testing was conducted through hand-searching the reference list of known peer-reviewed studies [12–17, 28–30] and an assessment of known grey literature [25, 27, 31, 32] and confirmed the appropriateness of this search strategy limitation.

The reference lists of articles that meet the inclusion criteria will be hand-searched. A forward citation search will be conducted using Google Scholar as this platform does do not rely on traditional indexation methods and is therefore better able to identify early online publications.

As required by the Preferred Reporting Items for Systematic Reviews and Meta-Analyses Protocol (PRISMA-P) guidelines [33], an illustrative search is documented for one database (Table 1).

**Table 1:** Example search for MEDLINE database to identify health economic evaluation literature in orthotics and/or prosthetics

Search number	Topic	Search field	Search term
1		MeSH	Exp Artificial Limbs/
2		MeSH	Exp Amputation/
3		MeSH	Exp Amputees/
4		MeSH	Exp Orthotic Devices/
5		MeSH	Exp Cost-benefit Analysis/
6		MeSH	Exp Costs and Cost Analysis/
7		MeSH	Exp Economic Evaluation/
8		MeSH	Exp Quality-adjusted life years/
9		MeSH	Exp Dentistry/
10			(OR/1–8) NOT 9
11	Intervention	ti, ab, kw	prosthe* OR orthot* OR orthos#s OR splint* OR brace* OR bracing
12	Outcome—general health economic terms	ti, ab, kw	((economic ADJ1 (impact OR value OR factor* OR analysis OR cost OR evaluation*)) OR (cost* ADJ1 (health care OR hospital OR medical)))
13	Outcome—health economic evaluation type	ti, ab, kw	(cost* ADJ1 (util* OR effective* OR efficacy* OR effic* OR benefit* OR consequence* OR analy* OR minimi* OR comparison OR saving* OR breakdown OR lowering OR estimate* OR variable* or allocation OR control))
14	Outcome—health economic measure used	ti, ab, kw	(((value OR values OR valuation) ADJ2 (money OR monetary OR life OR lives OR costs OR cost)) OR (quality-adjusted life ADJ1 (year* OR expectanc*)) OR (quality adjusted life ADJ1 (year* OR expectanc*)) OR (QOLY* OR QALY* OR QALE*))
15			10 AND 11 AND (OR/12–14)
16	Exclusion	ti	[15] NOT (breast OR fracture* OR arthrop* OR angiop* OR transplant* OR heart OR aort* OR valve* OR stent* OR retina* OR ocular OR hernia OR erect* OR carcinoma OR disc OR (total OR replacement* ADJ2 (hip OR knee OR ankle OR joint OR vascular)) OR (implant* ADJ2 cochlear) OR (cost ADJ3 (walking OR energy OR metabolic)))
17			Limit 16 to English language

Field codes: *MeSH* National Library of Medicine Subject Headings, *Exp* exploded, *ti* title, *ab* abstract, *kw* keyword as identified by author(s)

### Data management

The search results will be exported to EndNote X8.2 (Thomson Reuters Inc.). Duplicates will be removed using the EndNote “find duplicates” feature and through hand searching given variation in spelling or punctuation often prevent automatic detection of duplicates. Full-text articles will be appended to each EndNote record for studies that meet the inclusion criteria or where full-text articles are required to determine eligibility.

All EndNote records will be exported to a Microsoft Excel spreadsheet using a custom-made Endnote output style capturing key bibliographic information (e.g. authors, year, journal title). The Excel file will be customised to allow for the recording of decisions regarding inclusion/exclusion, data extraction and recording the checklist items from the critical appraisal of each article. A separate tab will also be used to record the results of screening based, so as to populate the PRISMA search flowchart (Additional file 1).

### Selection process

Articles will be included if they met the following criteria:

1. Research type: original, peer-reviewed research;
2. Language: published in the English language;
3. Year of publication: published since 1 January 2000;
4. Intervention: externally applied *orthoses* or *prostheses* for any part of the human body as defined by the International Standards, ISO 9999:2016 (ISO 8549 1–3 prosthetics and orthotics - vocabulary) [34], and provided by a healthcare profession as defined in the Health Practitioner Regulation National Law Act 2009 [35] and regulated via the Australian Health Practitioner Regulation Agency (AHPRA) in Australia or recognised by the National Alliance of Self Regulating Health Professions (NASRHP) (https://nasrhp.org.au/);
5. Outcome: a *health economic evaluation*, defined as “the comparative analysis of alternative courses of action in terms of both their costs and consequences” [18].

The operational definition of orthoses or prostheses (i.e. the intervention) will exclude studies of internal devices (e.g. hip and knee replacements or heart valve replacements). Where there are simultaneous treatments forming a co-intervention, such that the study does not inform the cost-effectiveness of a discrete orthotic/prosthetic device or service, the study will be excluded (i.e. conservative management versus surgical management of repetitive strain injuries, where the conservative intervention is multi-faceted including combinations of bracing and physiotherapy). The intervention under investigation must be provided by a recognised health care profession as defined by AHPRA or NASRHP, in which orthotic and/or prosthetic services are accepted as within the practitioner’s scope of practice. This definition acknowledges the substantial overlap in the scope of practice for several health professions (e.g. occupational therapists and orthotists both provide upper limb orthotic services, and podiatrists and orthotists both provide foot orthotic services). The operational definition of health economic evaluation will limit the inclusion of studies to those using cost-effectiveness, cost-utility, cost-benefit, cost-minimisation or studies with multiple outcome measures (cost-consequence) methodologies, either as experimental trials or through simulation (i.e. a Markov model) [18, 20]. As such, studies describing the cost of the intervention (i.e. cost comparison studies) will be excluded.

Due to the unambiguous nature of the inclusion criteria which does not require complex judgement, the search yield will be screened by one investigator (LC) based on a review of the title and abstract [36]. Full-text articles will be retrieved and reviewed as necessary to determine inclusion. A second opinion from another investigator (MD) will be sought as required and any disagreement will be resolved through discussion until consensus. Where consensus cannot be reached, a third reviewer (AS) will be called upon to provide advice and resolve conflict as necessary.

### Quality appraisal and risk of bias

The Consolidated Health Economic Evaluation Reporting Standards (CHEERS) checklist [18] will be used to appraise quality of the reporting. In concert, the Consensus on Health Economic Criteria (CHEC)-Extended list [37, 38] will be used to assess risk of bias of individual studies.

The CHEERS checklist was developed by the International Society for Pharmacoeconomics and Outcomes Research (ISPOR) *Health Economic Evaluation Publication Guidelines Good Reporting Practices Task Force* to facilitate consistent and transparent reporting of economic evaluation research and represents the most up-to-date and accepted standards in this area [18]. Whilst several researchers have used the CHEERS checklist as a risk of bias appraisal tool, its purpose is to guide researchers, editors and peer reviewers in best practice reporting for health economic evaluations and was not intended to assess the quality of the method design or execution of the study [18].

The CHEC-Extended list is recommended for the assessment of risk of bias where the literature contains both model-based and clinical trial or observational studies [39], as is the case in orthotics and prosthetics literature. The CHEC-Extended list is supported by detailed guidelines outlining the value judgement for each criterion and would likely address the poor inter-rater agreement inherent in the original CHEC list (0.33 intraclass correlation co-efficient; 95% CI 0.07–0.71) [40].

Whilst both the CHEC-Extended list and the CHEERS checklist have a small number of criteria that overlap (e.g. the description of the study population, description of competing alternatives and the definition of the research question), they will be used in their entirety, as originally designed and endorsed through a Delphi consensus process [38].

### Data extraction and risk of bias assessment process

A data extraction spreadsheet (Additional file 2), developed in Microsoft Excel, will be used to systematically record details of the intervention and setting (e.g. target population, type of intervention, setting/data source), characteristics of the sample (e.g. age, sex, time since amputation/diagnosis), features of the method design (e.g. type of health economic evaluation, time horizon, perspective, discounting methods and structural assumptions) and the recording of results (e.g. benefit outcome, cost outcome, and the incremental cost-effectiveness ratio). This approach has been described in comparable health economic evaluation systematic reviews [37].

The spreadsheet will include fields as specified in the CHEERS checklist and the CHEC-Extended list to allow for systematic recording of the appraisal outcomes. To reduce errors and omissions, the spreadsheet will be intuitively set up. For example, detailed questions, decision rules and guidelines are available by “scrolling-over” the column heading. Pull down menus with standard responses will be used to aid consistent data entry. Commentary space will be available for both the CHEERS checklist and CHEC-Extended list to record notes and justify decisions within pre-determined headings (e.g. subjects, methods, assumptions).

As part of protocol development, the data extraction spreadsheet has been developed and tested on two studies from the known bank of orthotic and prosthetic health economic evaluation studies. During testing, it became evident that the CHEC-Extended list guidelines could be used more reliably with a supporting content-specific decision aid that provides examples to contextualise the guidelines relative to the specific area of healthcare under review (Additional file 3).

Data extraction and completion of the CHEERS and CHEC-Extended will be undertaken by a primary reviewer (LC). A second reviewer (MD) will confirm the accuracy of these data and the risk of bias and reporting assessment and disagreement resolved through discussion. Single data extraction results in substantial time savings and is reported to have similar error rates to duplicate data extraction and appraisal, with minimal impact on the final extraction outcome [41, 42]. Furthermore, the appraisal tools have published decision guidelines which are further supported by our context-specific decision aid with exemplars, which reduce the likelihood of error. As necessary, a third reviewer (AS) will be called upon to provide advice or resolve conflict using discussion until consensus. Where necessary, the authors of the original research will be contacted to clarify aspects of the method design, result and/or reporting.

### Data summary and reporting

Initial scoping of the literature indicated that there are few health orthotic/prosthetic economic evaluations, and those identified use a diverse range of study designs, outcome measures and patient populations. Whilst we anticipate this will make a meta-analysis inappropriate, this will be tested through the completion of the data extraction process and where feasible a meta-analysis will be conducted. Where meta-analysis is not feasible, then the findings from this review will be reported as a narrative. The narrative will outline common issues with the reporting and method design that introduce bias with a view to explain the underlying cause of heterogeneity and imprecision that reduce confidence in the results and limit the synthesis of outcomes across studies. A discussion of the extent to which the literature contributes to orthotic and prosthetic policy and investment decisions will also be provided.

## Discussion

To our knowledge, this is the first systematic review of health economic evaluations pertaining to all orthotic and prosthetic interventions and services.

This protocol has been purposefully designed to summarise the existing evidence and, in doing so, appraise the methodological approaches used and the quality of the health economic evaluations relating to orthotics and prosthetics. What we learn from this review will be used to guide further work in this area and design more rigorous health economic evaluations into the future.

## Additional files

Additional file 1: PRISMA-P checklist (DOCX 17 kb)

Additional file 2: Data extraction template (XLSX 56 kb)

Additional file 3: CHEC-Extended content-specific decision aid (orthotics and prosthetics) (DOCX 121 kb)

## Data Availability

Not applicable

## Abbreviations

CDSR: Cochrane Database of Systematic Reviews
CHEC: Consensus on Health Economic Criteria list
CHEERS: Consolidated Health Economic Evaluation Reporting Standards
CINAHL: Cumulative Index of Nursing and Allied Health Literature
CRD: Centre for Reviews and Dissemination
HEED: Health economic evaluations database
MeSH: Medicine medical subject heading
NHS: National Health Service
NICE: National Institute for Health and Care Excellence
PRISMA: Preferred Reporting Items for Systematic Reviews and Meta-Analyses
PRISMA-P: Preferred Reporting Items for Systematic Reviews and Meta-Analyses Protocol
