# Supplementary material for: Health economic evaluation in orthotics and prosthetics: a systematic review protocol
Source: Syst Rev. 2019 Jun 27;8:152. doi: 10.1186/s13643-019-1066-9 (PMC6595622; doi:10.1186/s13643-019-1066-9)
Supplement: Supplementary file 3 — CHEC-Extended content-specific decision aid (orthotics and prosthetics) (DOCX 121 kb) [file 13643_2019_1066_MOESM3_ESM.docx]

**CHEC-Extended List Guidelines (O&P):**

Specified appraisal decision aid and examples for orthotic and prosthetic health economic evaluations

** the assessment outcome provided in the O&P example (i.e., YES or NO) relates only to the component of the decision rule in the example. It should not be interpreted that the study used in the example meets all the decision rules for the specific question*

# [STUDY DETAILS (questions 1, 2 and 3):](#_STUDY_DETAILS_(Questions)

The Study Detail questions assess whether the study population, competing alternatives and research question has been adequately described and is appropriate.

These questions allow reviewers to determine whether the findings could be generalised to the community population, whether the alternatives use are appropriate for the typical clinical setting and whether the research question is well-defined and is appropriate to be answered through a health economic evaluation.

# [METHODOLOGY (questions 4, 5, 6 and 7):](#_METHODOLOGY_(Questions_4,)

The Methodology questions assess the appropriateness of the economic study design, the structural assumptions within models, the chosen time horizon and perspective.

These questions allow reviewers to determine whether the unique health economic evaluation methodological design features are appropriate to answer the stated economic and/or policy decision and whether bias has been introduced to the study based on the approaches taken.

# [COSTS (questions 8, 9 and 10):](#_COSTS_(questions_8,)

The cost questions assess the identification, measurement and valuation of the costs used in the study.

The costs are one of the two key inputs in a health economic evaluation and these questions allow reviewers to determine the validity of the sources and reliability of the data that is used to inform the health economic evaluation outcome. The reliability of the cost data can be compromised if not all costs are identified, or they are identified but not measured correctly, or they are identified and measured, but not valued correctly. For example the cost data is collected in 2009 and the benefit data is collected in 2012, therefore the costs need to be valued in the 2012 period, requiring an adjustment for inflation.

*For example, when assessing an article by Janssen (2014), it is noted that the cost of patient time for the brace group is not identified (question 8) and there are cost measurement errors with patient time for the NeuroMuscular Training group calculated based on 20 minutes per session however the sessions take 30 minutes to complete (question 9). Whilst the costs are valued appropriately as this is a side by side trial so costs are valued in the same time period as the benefits are measured (question 10), the incorrect identification of costs in the brace group and measurement error in the NMT group reduces the reliability of the data to inform a health economic evaluation.*

# [BENEFITS (questions 11, 12 and 13):](#_BENEFITS_(questions_11,)

Benefit is the second key input in a health economic evaluation and therefore the benefit questions mirror the previous cost questions and assess the identification, measurement and valuation of the benefits used in the study. A benefit can be either a change in utility, such as an improvement in HR-QoL or a change in effectiveness, such as a reduction in falls or improved participation.

These questions allow reviewers to determine the validity of the sources and reliability of the data that is used to inform the health economic evaluation outcome. The reliability of the benefit data can be compromised if the appropriate benefit for the study and each alternative are not identified, or they are identified but not measured correctly, or they are identified and measured, but not valued correctly.

*For example, when assessing an article by Brodtkorb (2008), it is noted that Health Related Quality of Life (HR-QoL) is used to measure the benefit in terms of Quality Adjusted Life Years (QALYs) (question 11). Whilst this is an appropriate measure of the benefit, it is not measured appropriately given the survey was not administered correctly; that is, only a small part of the survey was used, not the whole survey as originally designed (question 12). Further to this HR-QoL is not valued equally across both alternatives. For example, given the way the instrument was administered, participants were asked to recall what their HRQoL was like for one of the alternatives. For the other alternative, participants were asked to reflect on their current health state. This introduces variation in how the alternatives are valued (question 13).*

# [STATISTICAL ANALYSIS (questions 14, 15 and 16):](#_STATISTICAL_ANALYSIS_(questions)

The Statistical Analysis questions assess the appropriateness of the incremental analysis of costs and benefits, the discounting of costs and benefits, and how uncertainty within variables in the cost and benefit inputs and the structural assumptions are managed through sensitivity analysis.

These questions allow reviewers to determine whether appropriate heath economic evaluation statistical techniques have been employed to engender confidence in the results.

# [CONCLUSIONS (questions 17, 18, 19 and 20):](#_CONCLUSIONS_(questions_17,)

The Conclusion questions assess whether the conclusions are appropriate given the results, whether consideration is given to the generalisability of the results and whether conflict of interest and ethical and distributional issues are appropriately reported and discussed.

These questions allow reviewers to determine the strength of the conclusions drawn in relation to the results and whether the results valuably contribute to the health economic decision the study sought to answer.

# STUDY DETAILS (Questions 1, 2 and 3):

| **Is the study population clearly described? (Evers, 2005)** | | |
| --- | --- | --- |
| **Guidelines to support the value judgment (Odnoletkova, 2014)** | **O&P appraisal decision rules** | **O&P example** |
| The study population should be described in terms of geography, patient characteristics such as age, sex, ethnicity (Higgins et al.), co-morbid conditions, and disease stage/previous treatments, each of which should be appropriate to the decision problem. (Caro et al.)  The patient population to which the economic evaluation applies should be consistent with the patient population defined in the clinical part of the study (Cleemput et al.) | **YES** (all of the following):  there is sufficient detail of the study population to determine whether the study population is representative and which population the results of the study could be generalised to. As a minimum this must include:   - age (mean and SD), - proportion males/females, - cause of amputation/condition type (proportion of sample with different causes), - prevalence of common comorbid conditions (e.g., diabetes, renal disease, heart disease, retinopathy), - level of amputation/condition type (inc proportions as needed), - geographic characteristics - the study population is relevant to the decision problem, - the study populations are comparable:   - the study population for the clinical inputs (i.e., from which the benefits and cost were calculated) must be comparable to the population to which the economic evaluation is being applied   - the study populations for the competing alternatives must be comparable   - study population data may be presented in a table and appropriate statistical analysis conducted to determine any variance between the two populations   **NO** (any of the following):   - failure to address all the above items | *“The final sample included 70 C-Leg patients and 57 Micro Processor Knees (MPK) patients. Statistical analysis confirmed that the two groups were comparable, as no significant differences emerged in terms of demographics (i.e. age, gender, height, and weight), amputation, and socio-economic variables (e.g. civil and professional status, academic degree, hobby,*  *and sport) (Table 1).”* (Cutti, 2017)   - NO - This study does not report co-morbid conditions within the table   *“The costs of semirigid ankle brace for a six-week intervention was obtained*  *from Lardenoye et al. (2012) . . . Quality adjusted life year gained for a semi-rigid ankle brace was estimated from Cooke et al. (2009).”* (Fatoye, 2016)   - NO - This study does not report whether the study populations from which the modelling data is derived are comparable |

| 1. **Are competing alternatives clearly described? (Evers, 2005)** | | |
| --- | --- | --- |
| **Guidelines to support the value judgment (Odnoletkova, 2014)** | **O&P appraisal decision rules** | **O&P example** |
| The competing alternatives should be clearly defined in terms of frequency, component services, dose or intensity, duration, and any variations required for target subgroups.  It should be mentioned whether people involved in delivery of the intervention need to be trained (adapted from Higgins et al. and Caro et al.)  The choice of the comparator(s) should always be justified.  The comparator should be the most cost-effective alternative intervention currently available. | **YES** (all of the following):   - competing Orthotic and Prosthetic (O&P) alternatives are clearly described and in sufficient detail to allow a reader to cost the intervention in its entirety - the alternative O&P intervention is the most cost-effective alternative - the alternative O&P intervention is justified - training requirements (qualifications) or specific competencies to support delivery of the O&P alternatives are described   **NO** (any of the following):   - failure to address all the above items | For example, an O&P cost-effectiveness study must **describe** the brand, model and year, as well as other orthotic/prosthetic component and design features (e.g., type of prosthetic socket and liner, type of prosthetic knee and foot).  The competing alternative/s must be a **realistic alternative**, such as a hydraulic knee (e.g., not a safety knee) and include adequate description/definition to allow replication of the study or consideration of the costings  *“Two alternatives were compared: (1) knee prosthesis with complete microprocessor control (C-Leg), and (2) mechanical knee prosthesis (articulating polycentric type)”* (Gerzeli, 2009)   - NO – This study provides limited detail of the competing alternatives and does not provide sufficient detail of the intervention in its entirety (e.g., foot and socket).   *“The latter is considered to be the next-best alternative to C-Leg currently reimbursed by the Italian NHS. According to clinical protocol used by the INAIL centre, all trans-femoral amputees are candidates for C-Leg application* (Gerzeli, 2009)   - YES – justification for the competing alternative is sufficient.   *Most commonly supplied in the back-up prosthesis of C-Leg users . . . Only 3 MPK’s satisfied these criteria, that is 3R60, Total Knee 2100 and Total Knee 2000* (Cutti, 2017)   - YES – justification for the competing alternative is sufficient.   The competing alternatives are provided by appropriately qualified healthcare professionals (e.g., a qualified prosthetist). The qualification status or competencies required to deliver the alternatives must be stated.  *“All patients receiving taping treatment were required to visit their Physiotherapist or Nurse on average of once per week . . . . patients attending treatment sessions administered by Physiotherapist or Nurse, once every two weeks (bracing)”* (Fatoye, 2016)   - NO – implies that a qualified health care professional is delivering the competing alternatives, but this is not explicit and the qualification or competency is not stated. |

| 1. **Is a well-defined research question posed in answerable form? (Evers, 2005)** | | |
| --- | --- | --- |
| **Guidelines to support the value judgment (Odnoletkova, 2014)** | **O&P appraisal decision rules** | **O&P example** |
| The research question should specify the type of population (participants), type of interventions (and comparisons), the type of outcomes and the type of study that was performed (Higgins et al.) including the chosen perspective and the applied analytic time horizon. | **YES** (all of the following):   - research question includes population, competing alternatives, type of outcomes and type of Health Economic Evaluation (HEE) study, chosen perspective and the time horizon   **NO** (any of the following):   - research question fails to include all the above items | *“The aim of this study was to assess the cost-effectiveness and outcome of OI-prostheses compared with the use of routine S-prostheses for patients with unilateral Trans Femoral Amputation (TFA) treated with OPRA Osseointegration in Sweden”* (Hansson, 2018)   - NO - This aim does not detail the perspective or time horizon |

# METHODOLOGY (Questions 4, 5, 6 and 7):

| 1. **Is the economic study design appropriate to the stated objective? (Evers, 2005)** | | | |
| --- | --- | --- | --- |
| **Guidelines to support the value judgment (Odnoletkova, 2014)** | **Specific guidelines for models (Odnoletkova, 2014)** | **O&P appraisal decision rules** | **O&P example** |
| Trial based economic evaluations are appropriate when the available data are sufficient to allow a full assessment of the cost-effectiveness or cost-utility of an intervention.  That means:   - the benefits of the treatment cannot go beyond the duration of the trial; - the intermediate benefits parameters do not have potential impact on the clinical endpoints such as long- term mortality, quality adjusted life years gained or life years gained on a long term (adapted from Cleemput et al.) | Modelling should be applied if the available data are insufficient to allow a full assessment of the cost- effectiveness or cost-utility of an intervention.  That means:   - the effect of the treatment might go beyond the duration of the trial; - the intermediate benefit parameters have potential impact on the clinical endpoints such as long-term mortality, quality adjusted life years gained or life years gained on a long term.   Modelling is also appropriate:   - to simulate the real-life application of an intervention based on the data available from clinical trials. This can be done e.g. by adjusting for differences in baseline risk between the trial population and the real- world target population and adjusting for protocol-driven costs or events; - to account for possible externalities associated with the disease or treatment (e.g. transmission of infections, bacterial resistance…) that were not part of the original study design and therefore not captured during clinical trial; - to compare the intervention with the relevant comparator if the respective interventions have never been directly compared in a clinical trial.   (adapted from Cleemput et al.) | **YES** (one of the following):   - all costs and benefits are able to be captured within the study period (Trial) - costs and benefits extend beyond the study period (Model)   Further definition:   - Trial – the effect of the intervention is limited to the study time period and doesn’t extend beyond or sufficient data is available within the study time period to inform the cost-effectiveness given the perspective taken by the study - Model – appropriate if treatment benefit extends beyond what a trial can capture, or where the respective interventions have never been directly compared or the costs and consequences not appropriately captured   **NO** (any of the following):   - A trial design is used, but the costs and benefits extend beyond the trial period and therefore are not appropriate captured (e.g., a societal perspective is taken) - A model design is used, but the costs and benefits of the competing alternatives are short-term or previously compared in a clinical trial (e.g., costs and benefits data exist) | A trial is appropriate to measure cost-effectiveness of short-term/acute interventions, such as ankle-sprain management, due to the short-term nature of the benefits. In the case of longer term/chronic management a trial may still be appropriate depending on the perspective taken.  The study design may also be influenced by the perspective that is taken. For O&P services for populations with chronic conditions where a societal perspective is taken, a model design is required to capture the long-term benefit.  However, if a healthcare payer perspective is taken then the benefit may only need to be captured for the life cycle of the competing alternatives under investigation, which may be short (trial) or long (model).    *“The analysis was undertaken from a Swedish health care perspective including costs directly associated with the health care provider. . . . The time horizon for the analysis was 8 years from the delivery of the first prosthesis because this is the functional time of a C-Leg guaranteed by the manufacturer”* (Brodtkorb, 2008)   - YES – Model design is appropriate and justified as the costs and benefits extend beyond a typical clinical trial period and the life cycle of the alternative is defined and links to the Funder (Health care payor) perspective |

| 1. **Are the structural assumptions and the validation methods of the model properly reported? (Evers, 2005)** | | |
| --- | --- | --- |
| **Specific guidelines for models (Odnoletkova, 2014)** | **O&P appraisal decision rules** | **O&P example** |
| For models, the following information should be presented:  Structure   - the structural hypotheses/ assumptions, - the uncertainty around these assumptions, - sources of information for these assumptions (systematic reviews preferred).   (adapted from Cleemput et al.)  Validity  Methods to verify the model’s   - structure (face) validity, - performance (technical/internal) validity - outcomes validity should be discussed.   (adapted from Weinstein et al. and Caro et al.)  **Supporting definitions:**  **Face validity:** Does the model reflect a clinically realistic scenario? e.g., is the life span of the MPK realistic? Are the costs of labour, number of hours, or service intervals for the intervention realistic?  **Internal validity:** are the structural assumptions of the study valid? e.g., are the competing alternatives provided in a manner which is appropriate to the interventions being compared?  **Outcomes validity:** likelihood that the outcome corresponds accurately to the real-world. Is the outcome measure appropriate for use in this population and research question? e.g., is the QoL instrument used appropriate for use in an amputee population? | **YES** (all of the following):  Structural assumption detail must include:   - the boundaries of the model (time, inclusions) and the events occurring (including their timing) reflect typical practice/pathways, - how the cycle length was determined, including a reference to current practice or support by an evidence source, - the transition probabilities (e.g., probability of movement between states) and the cycle length - how the transition probabilities were determined including a reference to current practice or support by an evidence source, - whether the transition probabilities are fixed for every cycle (i.e., a Markov chain) or do they vary for each cycle and an explanation.   The validity detail must include:   - the uncertainty is characterised/fully expressed (often using probabilistic sensitivity analysis or deterministic sensitivity analysis - one-way or multi-way) for the parameters (estimates of the inputs) and the structure (scientific judgements made in developing the model), - internal validity is verified, - the impact of the sensitivity analysis on the validity of the results discussed.   (Drummond, 2015, p.326-327; p.332-33; p.390-92)  **NO** (any of the following):   - insufficient detail in the above areas to provide confidence that the structural assumptions have resulted in the development of a model that reflects typical practice/pathways and real-world experiences.   **N/A:**   - For clinical trial studies, that do not use modelling | *“When fitting patient’s with a C-Leg, the patient’s are provided with a prosthesis and guaranteed a working knee . . . This service plan is offered for 8 years, after which the prosthetist is recommended to change the knee for a new unit because the cost of retaining the guarantee for the ninth year is a quarter of that of a new knee”* (Brodtkorb, 2008)   - YES – the study outlines how the boundaries of the model and cycle length were determined, referencing current practice.   *“Assumptions: First-time acute ankle sprains healing takes between 6-12 weeks (Hubbard, 2008) . . . assumed that one tape is used for taping an ankle per week (Olmsted, 2004) . . . One semi-rigid ankle brace was used for either the 6 week or 12 week period”* (Fatoye, 2016)   - YES – the assumptions underpinning the timing are provided and evidenced   Complete example:  the model time period (e.g., 3 months) relates to known time for ankle sprain healing, all likely events are included that reflect common treatment (e.g., at one month, possible events could be; continuing to heal, re-sprain) and is evidenced, the cycle length is short (e.g., each month) to reflect quick changes in state for acute injuries, the transition probabilities between states based on a Systematic Review of clinical outcomes for treatment for ankle sprains, the transition probabilities are not fixed as re-occurrence is possible.  The parameters within this model, such as labour costs, are subjected to sensitivity analysis as they are based on expert opinion, to explore the impact of uncertainty on the results. Transition state probabilities, including re-occurrence of ankle sprain is also subjected to sensitivity analysis due to the high cost attached to it and likely impact on the results. |

| 1. **Is the chosen time horizon appropriate in order to include relevant costs and benefits? (Evers, 2005)** | | |
| --- | --- | --- |
| **Guidelines to support the value judgment (Odnoletkova, 2014)** | **O&P appraisal decision rules** | **O&P example** |
| The chosen time horizon should be long enough to capture relevant differences in benefits across strategies (Caro et al.).  Treatments of chronic diseases mostly have consequences over a patient’s lifetime (Cleemput et al.). | **YES:**   - the time horizon is long enough to capture all relevant costs and benefits of the competing alternatives - the time horizon is equal for costs and benefits if these are to be combined into a ratio (ICER) - the time horizon is justified   **NO:**   - the time horizon does not allow all costs and benefits to be captured. This is likely where a healthcare payer perspective is taken. | For example, a short time horizon following dysvascular amputation may not allow likely re-amputations (and associated costs) to be captured. Or, a short time horizon following provision of an MPK may not capture the substantial cost of replacement once the warranty period ends.  *“The time horizon chosen in this study was 12 weeks as healing time for first-time acute ankle sprains is between 6-12 weeks (Hubbard, et.al, 2008)”* (Fatoye, 2016)   - YES – sufficient justification, including referencing is provided for the chosen time horizon, also considering that a NHS perspective (Funder – Healthcare payor) is taken.   *“Total ongoing prosthetic care costs for SSP and BAP were estimated for the same participant for an overall duration of 12 years including a time horizon of 6 years before and after treatment, respectively. . . . studies reported SF-36 data collected within 6 months before (SSP) and 24 months after (BAP) treatment . . . inconsistency in time horizon (i.e., 6 years for cost, 2 years for QALY)”* (Frossard, 2018)   - NO – the time horizon is not equal for costs and benefits and an ICER from the inputs was calculated. All benefits of the competing alternatives may not have been captured within the 2 year horizon |

| 1. **Is the actual perspective chosen appropriate? (Evers, 2005)** | | |
| --- | --- | --- |
| **Guidelines to support the value judgment (Odnoletkova, 2014)** | **O&P appraisal decision rules** | **O&P example** |
| The perspective of the analysis should be stated and defined.  Analyses which take a perspective narrower than the societal perspective should report and justify the included and excluded outcomes. (Caro et al.) | **YES** (all of the following):   - the perspective is stated, defined and justified, - the perspective is appropriate for the aim of the study and those who the study seeks to inform (e.g., third-party payors) - if a societal perspective is NOT taken, the reason is justified   **NO:**   - the above requirements are not met | As a general rule, the perspective should reflect those who are intended to be informed by the analysis, in which case the focus should be on the decisions that are within their remit and the costs and consequences that are relevant to them.  If the research is to inform disability policy decisions, then it is reasonable that a narrower perspective would be taken and the societal impact of service not considered.  Types of perspectives that may be stated and defined include:   - Societal - Funder (Healthcare Payor) - Patient   For example, in a HEE comparing MPK and Non-Micro Processor Knees (NMPK), the perspective is commonly that of the healthcare payor (e.g., insurer) given the need for research showing the intervention is effective given the cost outlaid by the payor. The payor may not have a primary concern for the benefits associated with a societal perspective (e.g., increased participation in paid employment, ability to care for family) as it is likely this doesn’t affect their policy and reimbursement decisions.  *“The economic evaluation was performed from a societal perspective with a time horizon of 12 months. A societal perspective considers all relevant costs and effects, regardless of who pays or who benefits from the effects . . . The main outcome of the cost-effectiveness evaluation was injury reduction”* (Janssen, 2014)   - YES – the perspective is stated (societal), defined (what is considered and included) and loosely justified. As the main outcome is injury reduction and the time horizon is short, a societal perspective would be expected. |

# COSTS (questions 8, 9 and 10):

| 1. **Are all important and relevant costs for each alternative identified? (Evers, 2005)** | | |
| --- | --- | --- |
| **Guidelines to support the value judgment (Odnoletkova, 2014)** | **O&P appraisal decision rules** | **O&P example** |
| The identification of costs should be consistent with the chosen perspective and the assessed area of disease and treatments.  For the perspective of the health care payer, at least all direct health care costs associated with or influenced by the competing alternatives must be included.  For the societal perspective, also direct and indirect costs outside the health care sector, such as productivity loss, should be included.  (adapted from Cleemput et al.) | **YES:** (all of the following):   - all direct costs are identified - all indirect costs are identified (if a societal perspective is taken) - costs included/excluded are justified   **NO:** (any of the following):   - the above requirements are not met - the costs are not appropriate for the perspective taken   (Drummond, 2015, p.46-48) | Examples:  Direct costs – materials, components, labour, repairs, rent, depreciation on equipment, additional clinical services. However, if a payor perspective is taken then it is assumed that many of these direct costs are captured in the purchase price of the service/competing alternative. Justification of inclusion/exclusion must be provided  e.g., inclusion of component, materials, clinical labour, administration etc should be detailed and included for O&P services  *“The annual cost of components of OI- and S-prostheses included the cost of the initial components and the requirements of new prostheses, servicing, repairs, adjustments, and maintenance which consisted of workshop salaries, investments in equipment and buildings, and consumer goods based on the cost reported previously by Haggstrom et al. (17)”* (Hansson,2018)   - YES – an example of appropriate identification and justification of the direct costs related to O&P interventions. Noting the article also identifies and justifies the direct costs associated with health utilisation for the OI-prosthesis alternative.   Indirect costs – loss of productivity, carer support, additional health care costs incurred. If a societal perspective is taken, then loss of productivity, reliance on informal supports and healthcare usage are examples of indirect costs which should be considered.  *“. . . participants received a cost diary in which they were asked to continuously register all absence from work, school, and other chores of life and all health care utilisation (including the use of medication) from the moment of injury until full recovery. This approach is recommended (9) and equal to the methodology applied in previous studies on this topic (11, 27)”* (Janssen, 2014)   - YES – a societal perspective is taken, and appropriate indirect costs are identified and justified |

| 1. **Are all costs measured appropriately in physical units? (Evers, 2005)** | | |
| --- | --- | --- |
| **Guidelines to support the value judgment (Odnoletkova, 2014)** | **O&P appraisal decision rules** | **O&P example** |
| Validated sources should be used for the measurement of the resource – and material use, such as observations from clinical trials, prospective observational studies, databases and patient charts, or derived from literature. If derived from literature or studies from other countries, resource use estimates should be validated for the local context (Cleemput et al.). | **YES:** (all of the following):   - costs are measured appropriately in physical units, - costs are measured using valid sources, - where the costs are from the literature or other countries, they are validated for the local context   **NO:**   - costs are not measured using valid sources   (Drummond, 2015, p.46) | Valid sources:  Costs extracted from the literature, clinical trials, observational studies, databases or financial records.  *“The actual costs were extracted from a total of 880 claims corresponding to a single item of expense including 569 for SSP and 311 for BAP, respectively. These costs are aggregated in Table 2”.* (Frossard, 2018)   - YES – costs are measured in appropriate physical units and extracted from a valid, local source   Validated for the local context:  The validity of the resource use for the HEE is described. For example, the study details the similarities between service models for two countries, such that clinical and technical resource use would be comparable, and the costings of one country are valid for use in the HEE in the other setting.  *“Finally, in the absence of local data, . . . costs of post minor and post major amputations were informed by a Swedish study. As the costs of post minor and major amputations are not only from another country but also from another era in health care, it is possible that the long-term costs of living with an amputation may have been underestimated in the current study. Compared with the Swedish study (26) . . . one Australian study reported lower costs of amputation (minor and major amputations combined) (54), while one US study reported higher costs of post minor and major amputations (55)”.* (Cheng, 2017)   - YES – the authors describe the limitations of the cost resource and details the likely applicability of the results and further outlines the use of probabilistic sensitivity analysis to manage the uncertainty with the cost estimates   Non-validated sources:  Costs measured through patient recollection, practitioner estimations etc, which introduce recall bias.  *“The resources used that were associated with the different health states and transitions were obtained from the interviews with the prosthetists and from manufacturers of the prosthetic components . . . The rates and the duration of the problem were elicited from the interviews with the patients. . . . The additional costs acquired when having a problem were elicited from the interviews with the prosthetists.”* (Brodtkorb, 2008)   - NO – costs are not measured using valid sources but through patient and prosthetic recollection of the resource use and rates of problems, from which costs estimates were established. |

| 1. **Are costs valued appropriately? (Evers, 2005)** | | |
| --- | --- | --- |
| **Guidelines to support the value judgment (Odnoletkova, 2014)** | **O&P appraisal decision rules** | **O&P example** |
| All costs should be expressed in values by using prices of a particular year indicated in the study (adapted from Cleemput et al.).  Adjustment for inflation should be based on the Consumer Price Index (CPI) or its health-care component.  The method of choice for making adjustments across countries is to use purchasing power parity. However, a simple currency conversion would be appropriate if there is an international market for an input at a fixed price (Weinstein et al.) | **YES:** (all of the following):   - the year the cost was borne is cited, - CPI is applied and described to bring costs in line with the benefit time period where required, - currency conversion is undertaken to align costs with the study population where required   **NO:**   - the above requirements are not met | For example, where a cost comparison study is used to provide cost data, the appropriate currency conversion and CPI must be applied.  *“As there are currently no Australian data available on the cost of post minor amputation and post major amputation care in the community, these costs were informed by a Swedish study (26) and were converted to AUD (average exchange rates in 1998: 1 SEK – 0.2 AUD) and then inflated to 2013 prices (47)”* (Cheng, 2017)   - YES – The cost was borne in 1998 in Sweden and converted to AUD using 1998 exchange rate and then CPI applied to 2013. |

# BENEFITS (questions 11, 12 and 13):

| 1. **Are all important and relevant benefits for each alternative identified? (Evers, 2005)** | | |
| --- | --- | --- |
| **Guidelines to support the value judgment (Odnoletkova, 2014)** | **O&P appraisal decision rules** | **O&P example** |
| Benefits in economic evaluations should be expressed in terms of final endpoints instead of intermediary benefits, i.e. in life years gained, in quality adjusted life years (QALYs) gained (Cleemput et al.), or in disability-adjusted life-years. (Caro et al.) Intermediate benefits (useful for benefit validation) can of course be reported and may include number of events, incidence of disease, mortality, adverse events … (Caro et al.). Differences in benefits between subgroups should be stated if appropriate (Cleemput et al.) | **YES:** (all of the following):   - the benefits identified for each O&P competing alternative are described and are relevant to the aim of the study or the purpose of the competing alternatives being compared, - the benefits represent end-points or appropriate intermediate benefits   **NO:**   - the above requirements are not met | For example, QALYs, disability-adjusted life years are an appropriate “end-point” measurement for O&P interventions.  In contrast, the number of falls, a measure of participation, or prevention or ankle sprains (Janssen, 2014) would be considered appropriate “intermediate benefit”. Intermediate benefits refer to a measurement point after which other benefits (such as HR-QoL, death) may continue to occur.  *“ICER presents the incremental costs of either the NMT or brace group to prevent a recurrence of ankle sprains in comparison with the combination group”.* (Janssen, 2014)   - NO – recurrent ankle sprain is an appropriate intermediate benefit, with reduction in the number of recurrent ankle sprains the measure of benefit, for the competing alternatives in this study, however “recurrence is not operationally defined.   *“We estimated participant utilities by administering the EQ5D instrument at baseline and 16 weeks. . . . no statistically significant effect if the intervention on QALYs was found. . . . Both arms showed minimal improvement over the course of the study, although this may reflect a lack of sensitivity of the EQ5D to pick up subtle disease changes in the RA foot, which may have been masked by overall disease activity”.* (Rome, 2017)   - NO – HR-QoL is not a relevant benefit for this study given the 16 week time horizon and the purpose of the intervention. Of note, pain scores improved significantly for both competing alternatives and the Foot Function Index score had a statistically significant improvement for one of the competing alternatives, suggesting an intermediate benefit may have been more appropriate. |

| 1. **Are all benefits measured appropriately? (Evers, 2005)** | | |
| --- | --- | --- |
| **Guidelines to support the value judgment (Odnoletkova, 2014)** | **O&P appraisal decision rules** | **O&P example** |
| Quality of the clinical evidence from which the differences in health benefits were derived, should be critically appraised.  QALYs should be derived from the utility weights obtained from the self-reported health status of the study participants. Validated generic health-related quality-of-life instruments should be used. | **YES:** (all of the following):   - The health benefit evidence is of sufficient rigour, including where benefit data is derived from previous studies - QALYs are derived utility weights obtained from self-reported HR-QoL assessments, in which:   - a validated, generic HR-QoL instrument (or instrument for intermediate benefits) is described and used,   - the validated generic HR-QoL instrument is administered correctly, in line with validated protocols   **NO:**   - the above requirements are not met | Instruments with established validity (e.g., SF-36, WHO QOL) will be considered valid even if not yet validated on populations requiring O&P interventions. The instrument must be used in its entirety as originally designed and validated. Where isolated subscales are used, the instrument must have been validated for use in discrete subscales.  Intermediate benefits refer to a measurement point after which other benefits (such as QoL) may continue to occur, e.g., number of falls, reduction in pain.  *“The utility weights needed to calculate the QALYs in the analysis were obtained from the interview with the patients. The patients were asked to assess their current QOL and estimate their QOL given they had not had the C-Leg. The rating scale in the EuroQol visual analog scale (VAS) was use for these assessments”.* (Brodtkorb, 2008)   - NO – the HR-QoL instrument is incorrectly administered, using only the VAS rather than the complete, validated tool. Further to this, recall bias is introduced as the EuroQol is not administered according to tool protocol as patients are required to “estimate” HR-QoL for the non-C-Leg competing alternative. |

| 1. **Are benefits valued appropriately? (Evers, 2005)** | | |
| --- | --- | --- |
| **Guidelines to support the value judgment (Odnoletkova, 2014)** | **O&P appraisal decision rules** | **O&P example** |
| The generic health-related quality of life instruments used should correspond with pre-specified scoring systems based on “forced-choice” methods (standard gamble, time trade-off) reflecting the preferences of the general public (Weinstein et al.).  Only if measured with the same instrument and in a similar patient population are the values comparable and can they be used in one and the same economic evaluation.  If the primary data are not available but only health-related quality-of-life results from trials from another country are used, index values from that country should be used for consistency. Adjustment for baseline (age- and gender-specific) health- related quality of life is required in estimating the incremental utility of an intervention (Cleemput et al.).  Life expectancy should be estimated by using national life tables based on all-cause mortality (Weinstein et al. and Cleemput et al.). | **YES:** (all of the following):   - the generic HR-QoL instrument is administered across all competing alternatives, and the populations for both alternatives are comparable and, - index values to account for different countries and adjustments for baseline are made as appropriate   **NO:**   - the above requirements are not met | For example, HR-QoL data is drawn from a side-by-side clinical trial or previously published trial in which the researchers demonstrate that the competing alternatives populations are comparable. e.g., EQ-5D data for MPK and a NMPK is drawn from 2 separate studies but the study populations are demonstrated to be comparable.  Where the HR-QoL data is drawn from a trial from another country, index values are used to ensure the QALY derived from the HR-QoL data is locally valued and adjusted and consistent across both alternatives   - *the EQ-5D-5L single index system allows calculation of QALYs (*[*https://euroqol.org/wp-content/uploads/2016/09/EQ-5D-5L_UserGuide_2015.pdf - p.12*](https://euroqol.org/wp-content/uploads/2016/09/EQ-5D-5L_UserGuide_2015.pdf%20-%20p.12)*), and* - *the SF-36 data needs to be converted to SF-6D generic, preference based single index measure, using local valuations, as emerging evidence suggests health state values differ across countries (*[*https://www.sheffield.ac.uk/scharr/sections/heds/mvh/sf-6d/faqs*](https://www.sheffield.ac.uk/scharr/sections/heds/mvh/sf-6d/faqs)*)*   *“To measure utility, the survey included a standardised measure of QoL, the EQ-5D . . . By answering the five dimension items, each patient defines a health profile, for example 11121, that can be converted into an index score using an Excel sheet provided by the EuroQoL Group (25). Scores were computed by adopting the “UK EQ-5D index calculator” sheet, which returns values ranging from -0.594 (i.e. 33333) to 1.0 (i.e. 11111).”* (Cutti, 2017)   - YES – the outcome (HRQoL) is valued using the EQ-5D, including all 5 validated domains, and the UK index calculator is used to convert to an index score. |

# STATISTICAL ANALYSIS (questions 14, 15 and 16):

| 1. **Is an appropriate incremental analysis of costs and benefits of alternatives performed? (Evers, 2005)** | | |
| --- | --- | --- |
| **Guidelines to support the value judgment (Odnoletkova, 2014)** | **O&P appraisal decision rules** | **O&P example** |
| The difference in the relevant health benefits should be compared to the difference in all relevant costs associated with the competing alternative (i.e. not only the additional costs of the intervention). Consistency in the perspective and the time horizon of the clinical and the economic outcomes should thereby be pursued.  Incremental Cost-Effectiveness Ratios (ICER) should only be presented if the treatment is NOT dominant (lower costs and better effectiveness) or dominated (higher costs and lower effectiveness) (Cleemput et al.). | **YES:** (all of the following):   - incremental analysis presented as an ICER, where the difference in the costs are compared to the difference benefits - all relevant costs have been included for all competing alternatives (not just the additional costs between the alternatives) - there is consistency in the perspective and time horizon in the cost and benefit inputs - the ICER is only presented where an alternative is not dominant   **NO:**   - the above requirements are not met | **ICER** calculation =  Cost Difference (Cost A – Cost B)  Benefit Difference (QALY A – QALY B)  *“To measure utility, the survey included a standardised measure of QOL, the EQ-5D . . . The ICUR (Incremental Cost Utility Ratio) was calculated by dividing (1) the difference in costs and (2) the difference in utility between C-Leg and MPK competitors”* (Cutti, 2017)   - YES – the method of ICUR calculation is provided, where the benefits have been calculated from a QALY   *“The prosthesis consists of the socket, the knee and the foot. In the analysis however, only costs related to the knee (electronic or mechanic) were considered, since the other two components (socket and foot) are common to both alternatives”* (Gerzeli, 2009)   - NO - The ICER analysis presented in this article does not include all costs for all competing alternatives; which must occur even if the same components (e.g., prosthetic foot) are included in all competing alternatives*.*   *“Direct costs from the NHS perspective and from the participants’ perspective . . . using a health-care personal proforma completed at baseline and 16 weeks. . . . We estimated participant utilities by administering the EQ5D instrument at baseline and 16 weeks”* (Rome, 2017)   - Yes – consistency in the time horizon for the calculation of costs and utilities is provided   **Dominant alternative** is one where the cost is lower and benefit higher than the competing alternative.  *“The ICER of the brace group in comparison with the combination group was -€2828.30, based on a difference in the mean cost of -€76.16 and a difference in the mean effects of 2.68% . . . A total of 75% of the bootstrapped ICERs of the brace group are in the southeast (dominant) quadrant, indicating that, in the brace group, there were significantly lower costs and fewer recurrent ankle sprains . . . “* (Janssen, 2014)   - NO – a negative ICER indicates a dominant treatment, with lower cost and higher benefits, as also demonstrated in the tornado diagram in this article |

| 1. **Are all future costs and benefits discounted appropriately? (Evers, 2005)** | | |
| --- | --- | --- |
| **Guidelines to support the value judgment (Odnoletkova, 2014)** | **O&P appraisal decision rules** | **O&P example** |
| The method for discounting costs and health benefits to present value should be stated and justified (Weinstein et al.). | **YES**: (all of the following):   - the method for discounting future costs is stated and justified - the method for discounting health benefits to present value is stated and justified   **NO:**   - the above requirements are not met | *Discounting future costs and benefits:* *Discounting is used to ensure that the costs and the benefits are calculated for a common period.* *It accounts for the incurrence of immediate costs but the obtainment of benefits in future periods (e.g., a health care cost today could be saved (or invested elsewhere) and therefore worth more in a future state, reflecting a real rate of return). The longer the healthcare cost is delayed the less it costs, due to the investment return during that period. Therefore, costs need to be expressed in terms of their value in a common period, which is the present period, with their values discounted to present values, based on when they are incurred and a discount rate that reflects real rates of return.*  (Drummond, 2015, p.108-12)  *e.g., “The time horizon of this study was 12 months, and therefore, costs and effects were not discounted.”* (Janssen, 2014)   - YES – the approach to discounting both costs and effects is described and justified   *“Costs and QALYs were discounted by 3% per annum according to recent guidelines.”* (Brodtkorb, 2008)   - YES – the approach to discounting is described and supported by evidence and/or best practice guidelines |

| 1. **Are all important variables, whose values are uncertain, appropriately subjected to sensitivity analysis? (Evers, 2005)** | | | |
| --- | --- | --- | --- |
| **Guidelines to support the value judgment (Odnoletkova, 2014)** | **Specific guidelines for models (Odnoletkova, 2014)** | **O&P appraisal decision rules** | **O&P example** |
| For all economic evaluations, uncertainty should be analysed using appropriate statistical techniques.  For within-trial economic evaluations:   - The sample uncertainty should be presented through deterministic sensitivity analysis methods (point estimate and range). - Methodological uncertainty coming from the analytical methods chosen such as the discount rate/ missed data imputation etc. should be handled by one-way sensitivity analyses.   The incremental costs and incremental outcomes should be presented with the 95% confidence or credibility interval (Adapted from Cleemput et al.).  For all economic evaluations:  To assess the sensitivity of the results to the discount rate applied, different scenarios should be presented. For all analyses of data, methods to handle missing data should be described.  It is recommended to show the most important contributors to the uncertainty of the estimated incremental cost- effectiveness/cost-utility ratio (e.g. by means of a Tornado diagram).  The cost-effectiveness plane, with the results of the uncertainty analysis (such as Monte Carlo simulations or bootstrapping), should always be presented. In addition, if simulations are spread over different quadrants of the cost-effectiveness plane, the percentage of simulations in each quadrant should be reported (adapted from Cleemput et al.).  A cost-effectiveness plane should be displayed. The acceptability curve should be presented to show the probability that the treatment is cost-effective, given varying theoretical threshold values for the cost-effectiveness ratio (Cleemput et al.). | For models:   - The **parameter uncertainty** should be tested through probabilistic sensitivity analyses, e.g. by means of Monte Carlo simulations.   *Beta distributions are a natural match for binomial* *data; gamma or log normal for right skew parameters;* *log normal for relative risks or hazard ratios; logistic for odds ratios)*  *(Caro et al.)*   - The **structural uncertainty** should be tested through presenting different scenarios to show the impact of different extrapolation approaches on the results.   *Possible scenarios:*   1. *the treatment effect disappears immediately in the extrapolated phase (stop-and-drop approach);* 2. *the incremental treatment effect stays the same as during the observed phase;* 3. *the initial treatment effect fades out in thelong term.* | **YES:** (all of the following):   - different scenarios are presented to assess sensitivity to discount rate - management of missing data described - Tornado diagram to show contributors to uncertainty, with the cost-effectiveness plane (through Monte Carlo simulations or Bootstrapping), percentage of simulations in each quadrant and the acceptability curve should be presented   **AND,**  for within trial HEE:   - sample uncertainty via deterministic sensitivity analysis, - methodological uncertainty (discount rate, missed data) through one-way sensitivity analyses - incremental costs and outcomes presented with 95% CI   *e.g., the 95% CI presented for the SF-36 outcomes prior to calculation of the index rate (QALY)*  **AND,**  for model-based HEE:   - parameter uncertainty tested through probabilistic sensitivity analyses - structural uncertainty tested by presenting different scenarios   **NO:**   - the above requirements are not met | **Parameter uncertainty:** All variables in the analysis are potential candidates for the sensitivity analysis. Only variables that are certain or which have a minimal impact on the study results can be excluded from the sensitivity analysis. Furthermore, a justification should be given over the range of the variables used in the sensitivity analysis.  *e.g., varying component costs of prosthetic knee units, varying life of prosthetic knee unit, increasing stump revision rates for OI over time, varying benefit outcomes.*  It would be expected that researchers have run analyses showing the impact of modelling under these different scenarios (e.g., when the cost of the MPK is increased or decreased by 10% what is the effect on the ICER).  *“Because out method of collecting parameter values being more prone to bias than prospectively collected data, we performed 1-way sensitivity analyses for all parameters and three 2-way sensitivity analyses with parameters fixed at their 99^th^ percentile value most unfavourable to the C-Leg in addition to the probabilistic sensitivity analysis. . . . The sensitivity scenarios performed did not result in cost-effectiveness ratios that substantially would change the estimated cost-effectiveness of the C-Leg strategy. However the QOL associated with each prosthesis during the year appeared to have some impact on the cost-effectiveness.”*  (Brodtkorb, 2008)   - YES – the parameter uncertainty associated with the study design is managed through sensitivity analyses for all parameters   **Structural uncertainty:** The structure and assumptions within the model are tested by presented different scenarios, such as the use of Monte Carlo simulations  *“Analysis of uncertainty was performed in three parts. First, one-way sensitivity analysis was performed by varying 27 of the parameters in the model individually within an uncertainty range of ± 10% of the mean value. Secondly, probabilistic sensitivity analysis was undertaken to capture the uncertainty in all parameters simultaneously, using 5000 Monte Carlo samples. Parameter uncertainty was defined by probability distributions as recommended by Briggs et al.(29). Depending on the parameter, uncertainty was based on estimates of calculated or reported patient counts, standard errors, or range. Beta distributions were used for changes of probabilities and utility weights, and gamma distributions were used for costs.”* (Hansson, 2018)   - YES – Parameter uncertainty tested by varying 27 parameters and Structural uncertainty tested by completing 5000 Monte Carlo simulations.   *“As shown in the cost-effectiveness acceptability curve, there was a high probability of the C-Leg being cost-effective even for willingness to pay values well below conventional thresholds. However, such an analysis only considers statistical uncertainty surrounding the model input but does not relate to structural uncertainty, such as the omission of important events or cost item in the model. . . . One issue of structural uncertainty that was investigated was the analytic choice of a time horizon of 8 years instead of a lifetime perspective as generally advocated in cost-effectiveness analysis”.* (Brodtkorb, 2008)   - NO – parameter uncertainty is tested through sensitivity analysis, but structural uncertainty is only tested for one model assumption (time horizon) and the methodology for testing is not reported. |

# CONCLUSIONS (questions 17, 18, 19 and 20):

| 1. **Do the conclusions follow from the data reported? (Evers, 2005)** | | |
| --- | --- | --- |
| **Guidelines to support the value judgment (Odnoletkova, 2014)** | **O&P appraisal decision rules** | **O&P example** |
| Do the authors interpret their results cautiously and are their conclusions justified by the data (Evers, 2005).  Do the authors critically discuss the quality of health economic evidence considering study limitations and uncertainties? | **YES:** (all of the following):  conclusions are well supported by the results presented  the conclusions are fair given the limitations of the study (e.g., biases introduced in the design, or uncertainly in the costs) are fairly considered in the strength of conclusions  definitive conclusions are not made where there is uncertainly  **NO:**   - the above requirements are not met | For example, the authors draw their conclusions from the results and reflect on the impact of variances in costs or the effect of biases on the HEE result  *“The treatment effect of the intervention at 16 weeks was not significant between the two arms (p = 0.14). . . . there was a small statistically insignificant QALY loss associated with the CFMO intervention compared to SI. Therefore, no statistically significant effect of the intervention on QALYs was found . . . The CFMO was more expensive and less effective than the SI, therefore, is more dominated. . . . “* (Rome, 2017)  *“In people with established RA, semi-rigid customized foot orthoses can improve pain and disability scores in comparison to simple insoles. From a cost-effectiveness perspective, the customized foot orthoses were far more expensive to manufacture, with no significant cost per QALY gain.”* (Rome, 2017)   - NO – The first statement is from the Results and the second statement is the Conclusion in the Abstract. The conclusion, whilst not incorrect, is not representative of the key findings of the study. |

| 1. **Does the study discuss the generalizability of the results to other settings and patient/client groups? (Evers, 2005)** | | |
| --- | --- | --- |
| **Guidelines to support the value judgment (Odnoletkova, 2014)** | **O&P appraisal decision rules** | **O&P example** |
| Generalisability refers to applicability of the results to other populations (e.g. non-trial populations with different baseline risk). Transferability refers to the applicability of the results from other countries. These two aspects should be assessed separately (Cleemput et al.) | **YES:** (all of the following):  the authors reflect on the **generalisability** of the results to other populations  the authors reflect on the **transferability** of the results to other settings  **NO:**   - the above requirements are not met | The authors reflect on the **generalisability** of the results to the relevant population. For example, the authors consider the extent to which the results from the study sample can be generalised to people living in the community with the same condition and clinical presentation.  *“As detailed in Table 5 in Supplementary material, the main limitations and barriers to generalisation related to small convenient sample size, narrow case-mix, consideration of single reimbursement standards (ie., QALS), . . . differences in fixation types (i.e., costs for press-fit fixation, QALY for screw-type implant) discrepancy between perspectives . . . Altogether the generalisation of the study outcomes must be considered carefully giving (sic) the limitation and barriers presented in Table 5 in Supplementary material”.* (Frossard, 2018)   - YES – the authors reflect on and provide sufficient detail to support an understanding of the generalisability of the results. The lack of generalisability would be appraised through other CHEC+ questions, such as those exploring the competing alternatives, perspectives etc.   The authors reflect on the **transferability** of the results to other settings, such as overseas populations.  *“Finally, our research has context-specific elements, for example, the costs of prostheses, that might limit the direct application of the analysis in any context. We thus encourage replicating results across different national and local settings, as well as developing and exploiting multi-center databases”.* (Cutti, 2017)   - YES – the authors acknowledge the context-specific nature of the study (based in Italy) and encourage further research to support transferability of the results. |

| 1. **Does the article indicate/report that there is no potential Conflict of Interest (COI) of study researcher(s) and funder(s)? (Evers, 2005)** | | |
| --- | --- | --- |
| **Guidelines to support the value judgment (Odnoletkova, 2014)** | **O&P appraisal decision rules** | **O&P example** |
| No value judgment implied. | **YES:** (all of the following):  COI of researchers reported  COI of funders reported  **NO:**   - the above requirements are not met | If an external agency finances the study, a statement should explicitly be given about who financed the study to guarantee transparency in the relationship between the sponsor and the researcher. Whenever a potential conflict of interest is possible a declaration should be given of ‘competing interest’.  *“A commercial party having a direct financial interest in the results of the research supporting this article has conferred or will confer a financial benefit upon the author or 1 or more authors. Johannesen-Munk and Thidell financial support from Otto Bock for travel expenses associated with data collection. Brodtkorb has been a consultant to Otto Bock and competing prosthetic manufacturers.”* (Brodtkorb, 2008)   - YES – COI of researchers and funders reported |

| 1. **Are ethical and distributional issues discussed appropriately? (Evers, 2005)** | | |
| --- | --- | --- |
| **Guidelines to support the value judgment (Odnoletkova, 2014)** | **O&P appraisal decision rules** | **O&P example** |
| The morally relevant issues and moral conflicts related to implementing or not implementing the technology have to be synthesized and reported. This includes potential impact on the traditional values such as human equality, autonomy, dignity, the principles of solidarity and justice etc. Summarizing the benefits and harms of introducing/ refraining from the technology for different groups of stakeholders, such as patients, families, care providers, society etc. might be appropriate. The ethical analysis should allow a judgment on their transferability (adapted from The HTA Core Model) | **YES:** (all of the following):  The authors discuss ethical aspects and the implications  The authors discuss distributional issues  **NO:**   - the above requirements are not met | For example, the authors discuss the implications for the funding agencies and/or patients if MPK have been shown to be more cost-effective compared to NMPK knees? Can the funding agency afford the technology? If the funding agency does not approve the payment of the technology, what are the issues they face then in terms of liability or their ethical obligations?  *“The clinical implication is that, from a healthcare perspective, treatment with prostheses which are anchored to bone after a FTA results in improved quality of life at relatively high costs”.* (Hansson, 2018)   - NO – the ethical aspects and implications associated with the clinical implication statement are not explored. |

# References:

Brodtkorb TH, Henriksson M, Johannesen-Munk K, Thidell F. Cost-effectiveness of C-leg compared with non-microprocessor-controlled knees: a modeling approach. Archives of physical medicine and rehabilitation. 2008;89(1):24-30.

Cheng Q, Lazzarini PA, Gibb M, Derhy PH, Kinnear EM, Burn E, et al. A cost-effectiveness analysis of optimal care for diabetic foot ulcers in Australia. International wound journal. 2017;14(4):616-28.

Cutti AG, Lettieri E, Del Maestro M, Radaelli G, Luchetti M, Verni G, et al. Stratified cost-utility analysis of C-Leg versus mechanical knees: Findings from an Italian sample of transfemoral amputees. Prosthetics and orthotics international. 2017;41(3):227-36.

Drummond M, Sculpher M, Claxton K, Stoddart G, Torrance G. Methods for the economic evalution of health care programmes. New York: Oxford University Press; 2015.

Evers S, Goossens M, de Vet H, van Tulder M, Ament A. Criteria list for assessment of methodological quality of economic evaluations: Consensus on Health Economic Criteria. International journal of technology assessment in health care. 2005;21(2):240-5.

Fatoye F, Haigh C. The cost-effectiveness of semi-rigid ankle brace to facilitate return to work following first-time acute ankle sprains. Journal of clinical nursing. 2016;25(9-10):1435-43.

Frossard LA, Merlo G, Burkett B, Quincey T, Berg D. Cost-effectiveness of bone-anchored prostheses using osseointegrated fixation: Myth or

reality? Prosthetics and orthotics international. 2018;42(3):318-27.

Gerzeli S, Torbica A, Fattore G. Cost utility analysis of knee prosthesis with complete microprocessor control (C-leg) compared with mechanical technology in trans-femoral amputees. The European journal of health economics : HEPAC : health economics in prevention and care. 2009;10(1):47-55.

Hansson E, Hagberg K, Cawson M, Brodtkorb TH. Patients with unilateral transfemoral amputation treated with a percutaneous osseointegrated prosthesis. The bone & joint journal. 2018;100-b(4):527-34.

Janssen KW, Hendriks MR, van Mechelen W, Verhagen E. The Cost-Effectiveness of Measures to Prevent Recurrent Ankle Sprains: Results of a 3-Arm Randomized Controlled Trial. The American journal of sports medicine. 2014;42(7):1534-41.

Odnoletkova I. CHEC-Extended: A tool for the quality assessment of economic evaluations of healthcare interventions 2014.

Rome K, Clark H, Gray J, McMeekin P, Plant M, Dixon J. Clinical effectiveness and cost-effectiveness of foot orthoses for people with established rheumatoid arthritis: an exploratory clinical trial. Scandinavian journal of rheumatology. 2017;46(3):187-93.
